# Supplementary material for: Pentavalent HIV-1 vaccine protects against simian-human immunodeficiency virus challenge
Source: Nat Commun. 2017 Jun 8;8:15711. doi: 10.1038/ncomms15711 (PMC5472724; doi:10.1038/ncomms15711)
Supplement: Supplementary Information — Supplementary Figures [file ncomms15711-s1.pdf]

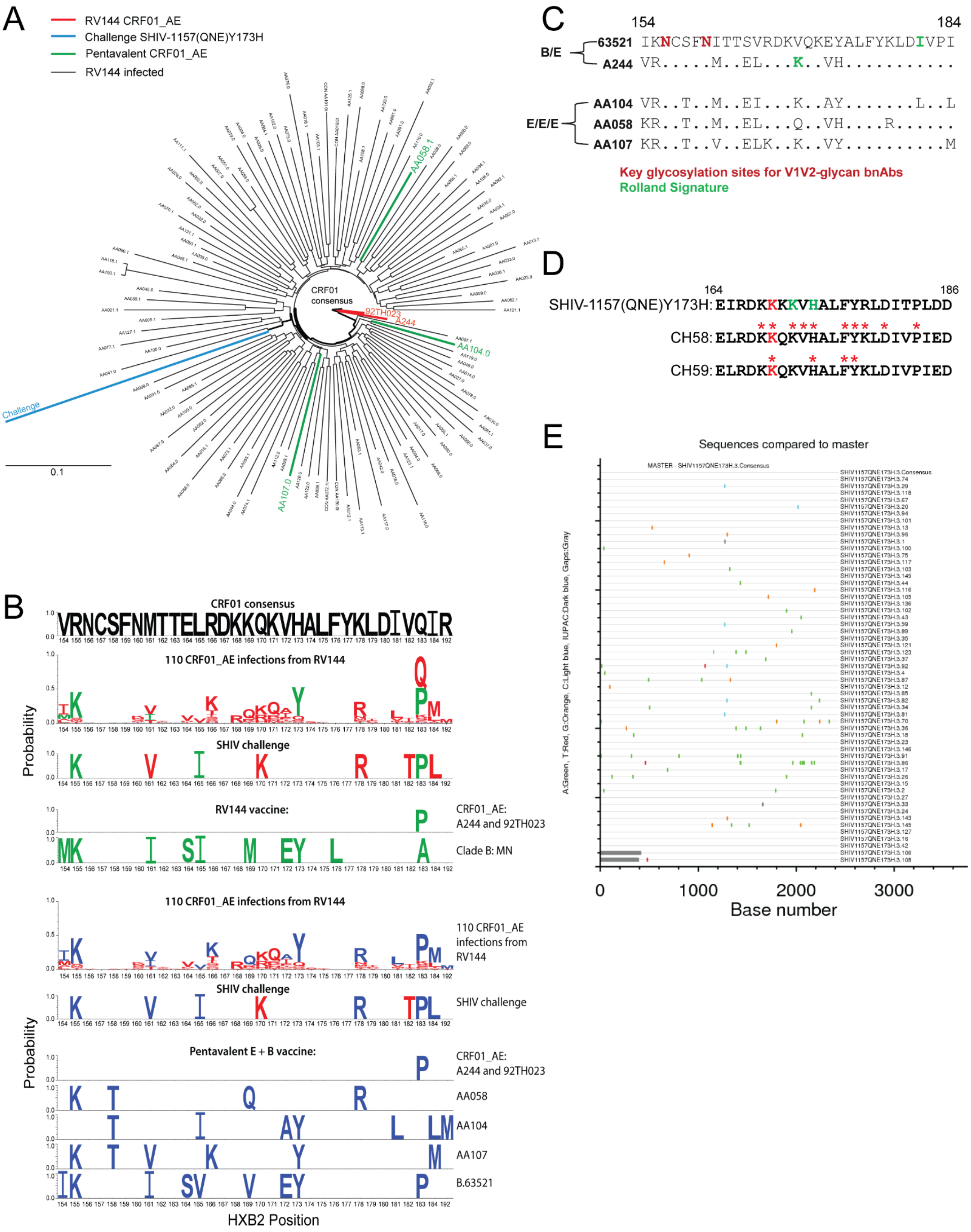
A
RV144 CRF01_AE
Challenge SHIV-1157(QNE)Y173H
Pentavalent CRF01_AE
RV144 infected
CRF01 consensus
92TH023
A244
AA058.1
AA104.0
AA107.0
Challenge
0.1
B
CRF01 consensus
VRNCSFNMTTELRDKKQKVHALFYKLDIVQIR
110 CRF01_AE infections from RV144
SHIV challenge
RV144 vaccine:
CRF01_AE: A244 and 92TH023
Clade B: MN
110 CRF01_AE infections from RV144
SHIV challenge
Pentavalent E + B vaccine:
CRF01_AE: A244 and 92TH023
AA058
AA104
AA107
B.63521
Probability
HXB2 Position
C
154
184
B/E
63521 IKNCSFNITTSVRDKVQKEYALFYKLDIVPI
A244 VR.....M..EL...K..VH..........
E/E/E
AA104 VR..T..M..EI...K..AY......L..L
AA058 KR..T..M..EL...Q..VH....R......
AA107 KR..T..V..ELK..K..VY.........M
Key glycosylation sites for V1V2-glycan bnAbs
Rolland Signature
D
164
186
SHIV-1157(QNE)Y173H: EIRDKKKKVHALFYRLDITPLDD
CH58: ELRDKKQKVHALFYKLDIVPIED
CH59: ELRDKKQKVHALFYKLDIVPIED
E
Sequences compared to master
MASTER - SHIV1157QNE173H.3.Consensus
A:Green, T:Red, G:Orange, C:Light blue, IUPAC:Dark blue, Gaps:Gray
0
1000
2000
3000
Base number

Supplementary Figure 1. ALVAC-protein vaccines and macaque immunization. (A) Maximum likelihood tree illustrating CRF01_AE gp120 protein sequence relationships between 107 Envs sampled in the RV144 trial (.1 vaccine and .0 placebo groups). The two CRF01_AE immunogens used in the RV144 vaccine are in red. The 3 additional natural Envs selected to be included in the pentavalent vaccine are in green, and the heterologous challenge SHIV (SHIV-1157(QNE)Y173H) shown in blue. **(B)** Logos comparing the vaccine antigens in the V2 region to the CRF01_AE sequences sampled from 110 infections in the RV144 trial and challenge SHIV. Black amino acids across represent the CRF01_AE consensus in this region and are not included in subsequent logos. Red amino acids are not found in the vaccines, RV144 vaccine residues are in green and pentavalent vaccine residues displayed in blue. The frequency of each amino acid in each position is indicated by the height of the letter on the y-axis, and HXB2 positions are highlighted from left to right. **(C)** Amino acid alignment of vaccine Env V2 sequences. Red are essential glycan sites and green residues are sites of immune pressure in RV144. **(D)** Alignment of amino acids of the challenge SHIV and A244 V2 sequence with contact sites for CH58 and CH59 indicated by the red stars. Site of immune pressure in RV144 are in red and green. **(E)** Single-genome amplification of the challenge virus stock. Nucleotide changes from the master sequence (top) are indicated by colored boxes.

A

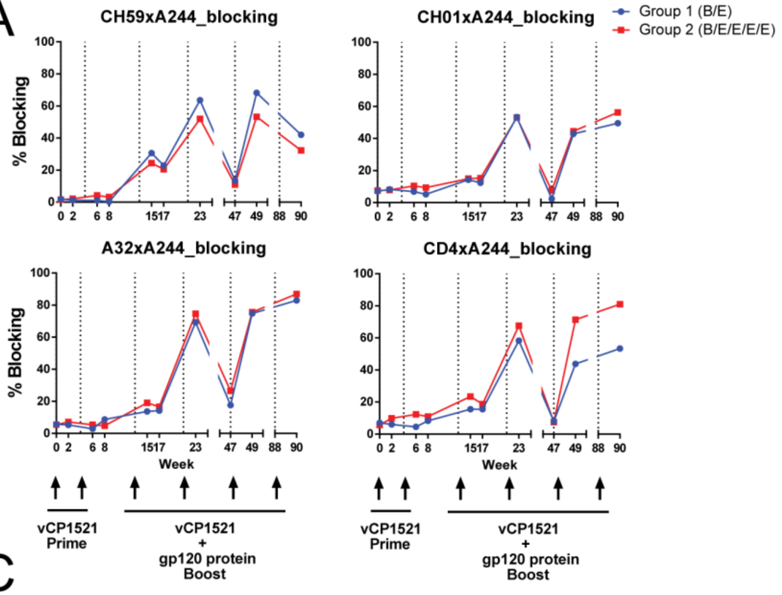

B

Epitope ID	Peptide Region
C1.68-84	#23-25
C1.83-100	#28-29
C1.95-121	#32-36
C1-V1.119-136	#40-41
V2.154-183	#50-55
V2.175-192	#57-58
C2.199-222	#65-68
C2.226-258	#74-80
C2-V3.289-303	#95
V3.298-321	#98-101
V3.312-335	#102-105
C3.354-375	#116-118
V4.405-423	#130-131
C4.424-453	#136-141
V5-C5.457-480	#147-150
C5.481-507	#155-159
C5.496-513	#160-161

C

Group	Animal ID	A Con	B Con	C Con	D Con	Group M	AE Con	AG Con	C ZM651	C TV1	C 1086	B MN	AE TH023	AE A244	All Clade*
1	201-08	8.0%	7.4%	10.4%	6.1%	6.7%	10.4%	6.1%	7.4%	8.6%	9.2%	8.6%	11.7%	10.4%	6.9%
1	35-12	6.7%	8.0%	4.9%	3.7%	5.5%	6.1%	6.7%	3.7%	4.9%	6.1%	9.8%	8.0%	5.5%	5.4%
1	36-12	3.7%	6.1%	4.3%	2.5%	3.7%	7.4%	3.7%	2.5%	7.4%	4.9%	5.5%	8.0%	7.4%	4.6%
1	47-12	9.2%	9.8%	12.9%	6.1%	8.0%	12.3%	11.0%	12.3%	12.3%	11.0%	12.3%	14.7%	15.3%	10.4%
1	92-09	3.7%	4.3%	6.1%	2.5%	4.3%	4.3%	4.9%	5.5%	6.1%	6.1%	5.5%	4.9%	6.1%	4.1%
1	43-12	6.7%	7.4%	8.0%	4.3%	5.5%	8.0%	6.7%	4.9%	7.4%	6.7%	8.0%	8.0%	8.0%	6.1%
1	53-12	6.7%	6.7%	9.2%	5.5%	5.5%	5.5%	4.9%	6.7%	6.7%	6.1%	6.7%	6.7%	8.6%	6.9%
1	71-12	4.3%	4.9%	3.1%	0.6%	3.1%	3.7%	4.3%	1.8%	3.7%	2.5%	5.5%	4.3%	5.5%	3.1%
1	86-12	1.2%	1.8%	4.3%	0.0%	1.8%	2.5%	1.2%	2.5%	5.5%	4.9%	2.5%	4.3%	3.7%	3.0%
2	357-08	6.7%	4.9%	4.9%	3.7%	4.9%	5.5%	6.1%	4.3%	5.5%	4.9%	5.5%	6.7%	6.7%	4.6%
2	41-12	6.1%	8.6%	7.4%	8.6%	6.1%	7.4%	6.1%	5.5%	9.2%	4.9%	9.8%	8.6%	5.5%	6.0%
2	49-12	3.1%	4.3%	4.3%	2.5%	3.1%	6.7%	3.7%	2.5%	7.4%	4.3%	5.5%	7.4%	6.1%	4.1%
2	54-12	8.0%	9.2%	11.0%	8.6%	6.7%	13.5%	8.6%	6.7%	9.8%	6.7%	9.2%	15.3%	14.7%	7.3%
2	63-12	3.1%	3.1%	3.1%	1.2%	1.8%	3.7%	2.5%	0.6%	4.3%	3.7%	3.7%	4.3%	3.1%	2.6%
2	77-12	6.7%	7.4%	8.0%	6.1%	6.7%	12.9%	7.4%	6.1%	8.0%	8.6%	7.4%	12.3%	10.4%	6.9%
2	52-12	9.8%	11.0%	16.6%	10.4%	12.3%	16.6%	11.0%	12.9%	14.1%	12.3%	11.7%	18.4%	17.2%	11.9%
2	74-12	7.4%	6.7%	8.6%	4.9%	6.1%	9.8%	7.4%	6.7%	9.8%	6.7%	8.0%	9.2%	9.8%	7.1%
2	48-12	12.3%	11.7%	14.1%	9.2%	12.9%	15.3%	11.7%	11.0%	13.5%	14.7%	12.9%	16.6%	16.6%	10.5%

D

Group	Animal ID	C1.1 #23-25	C1.2 #28-29	C1.3 #32-36	C1-V1 #40-41	V2 #50-57	C2.1 #65-68	C2.2 #74-80	V3 #95-106	C3 #116-118	V4 #130-131	C4 #136-141	V5-C5 #147-150	C5 #155-161
1	201-08	10,270	1	69	2,171	2,437	7,529	1	49,011	218	732	875	1,922	4,998
1	35-12	3,607	1	496	2,350	949	341	1	28,358	315	1	36	1,333	8,222
1	36-12	1,742	1	1	815	580	7,851	620	18,017	1	242	237	1,519	404
1	47-12	1,335	1	1,202	1	1,494	6,654	1	29,280	159	1	11,516	2,511	4,684
1	92-09	1	1	3,620	697	487	18,362	1	49,378	17	1	123	1	125
1	43-12	4	1	294	1	1	8,153	1	15,804	1	1	3,976	8,774	41,979
1	53-12	26,387	1	1	1,740	791	311	7,024	39,676	66	966	25,282	6,149	3,943
1	71-12	915	1	559	726	1	870	1	11,173	1	1	26	1,926	1,324
1	86-12	1	1	407	60	7,723	3,607	1	24,531	5,733	1	1	1	1
2	357-08	1	1	202	130	668	1,730	1	15,414	27	1	36	6,816	3,420
2	41-12	309	1	983	709	77	1	189	9,205	1	16	1	422	11,057
2	49-12	50	1	977	27	1	262	1	9,296	1	1	2,245	3,277	4,551
2	54-12	350	1	1,668	175	957	3,869	497	10,557	1	1	312	3,035	18,115
2	63-12	8	1	517	1	518	1	354	10,568	42	37	1	2,814	797
2	77-12	3,947	1	1,635	3,452	1	779	5,656	15,841	1	1	292	2,864	5,765
2	52-12	25,468	1	1,475	483	1,008	9,664	381	28,476	5,776	7	1,016	121	6,460
2	74-12	439	302	828	306	1	1,695	1,622	18,508	13	761	525	1,306	3,555
2	48-12	8,479	1	2,573	3,337	1,851	1,491	9,749	25,546	5,865	1	254	5,162	11,418

Supplementary Figure 2. Humoral immune response to vaccination. (A) Plasma antibody blocking of CH59, CH01, A32 antibodies and sCD4 to A244 over the course of immunization by competitive ELISA. Average of all animals graphed. **(B)** HIV-1 Env region/epitope and corresponding peptide number on array. **(C)** Chart showing the percentage of positive peptides in a peptide microarray that contains overlapping 15-mer peptides covering the Env gp160 for 7 clade consensus (A, B, C, D, M, AE, AG) and 6 vaccine strains (ZM651, TV1, 1086, MN, 92TH023, and A244). Positive threshold is signal intensity/IgG concentration > 100. **(D)** Chart showing the maximum binding intensity for all clades along the HIV-1 Env regions (MFI/IgG concentration).

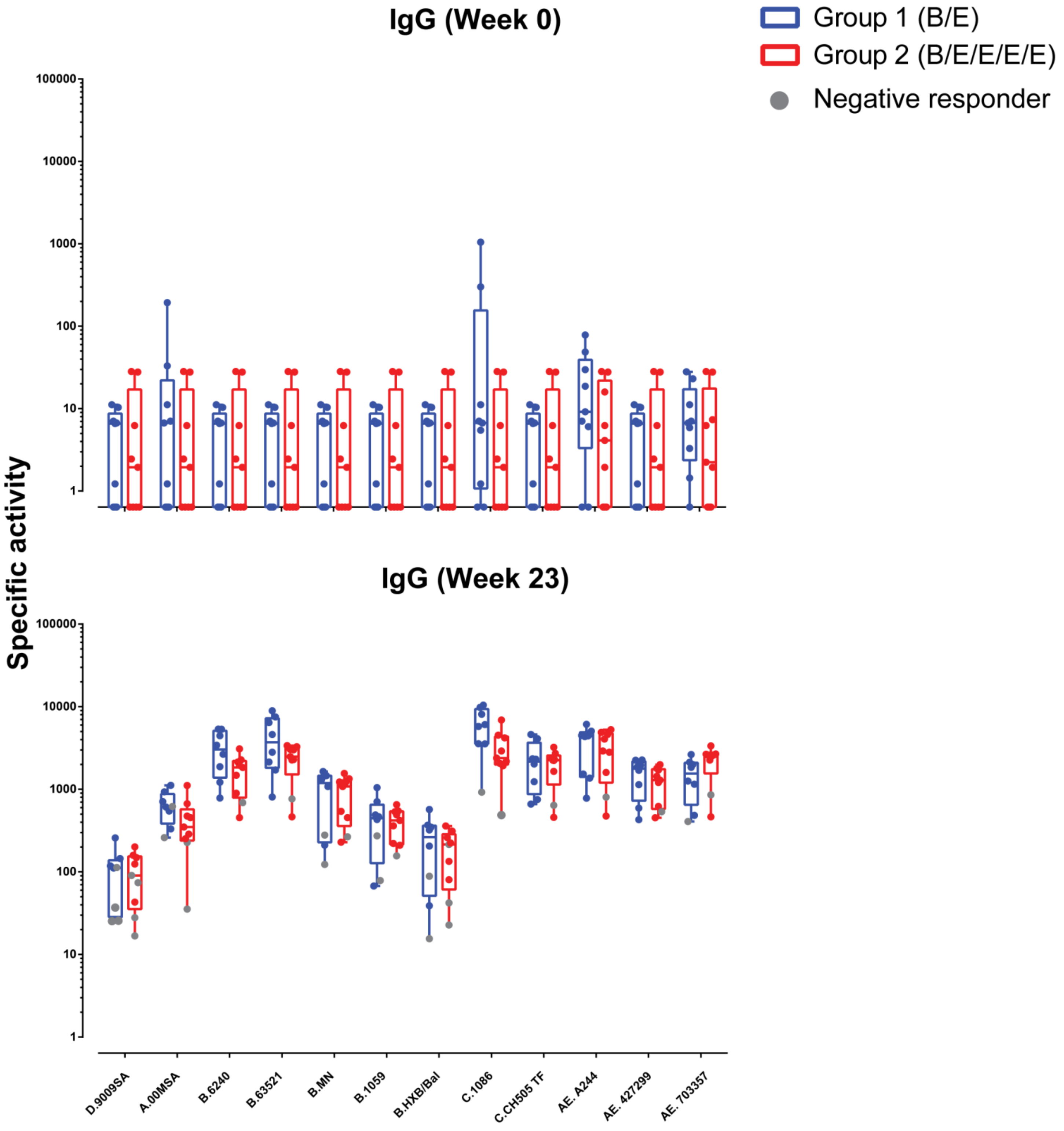

IgG (Week 0)
IgG (Week 23)
Group 1 (B/E)
Group 2 (B/E/E/E/E)
Negative responder
Specific activity
100000
10000
1000
100
10
1
D.9009SA
A.00MSA
B.6240
B.63521
B.MN
B.1059
B.HXB/Bal
C.1086
C.CH505 TF
AE. A244
AE. 427299
AE. 703357

Supplementary Figure 3. Mucosal antibody binding responses. Mucosal IgG purified from rectal wecks taken at week 0 and week 23 (after 2nd protein boost) were tested for binding of 12 multi-clade HIV-1 Env proteins using the binding antibody multiplex assay. Binding intensity measure as mean fluorescence intensity (MFI) and normalized by IgG concentration (specific activity). Positive response determined when specific activity 3x baseline and 95th percentile of all baseline samples for each antigen. Each dot within the box and whisker plot represents an individual animal in the bivalent (blue) and pentavalent (red) immunized groups.

A

			Group 1 (B/E)									Group 2 (B/E/E/E/E)								
Tier	Virus Name	Week	35-12	47-12	53-12	71-12	74-12	86-12	92-09	201-08	357-08	36-12	41-12	43-12	48-12	49-12	52-12	54-12	63-12	77-12
2	SHIV1157(QNE)Y173H	90	<20.0	<20.0	<20.0	<20.0	<20.0	<20.0	<20.0	<20.0	<20.0	<20.0	<20.0	<20.0	<20.0	<20.0	<20.0	<20.0	<20.0	<20.0
2	AA058a04R	0	<20.0	<20.0	<20.0	<20.0	<20.0	<20.0	<20.0	<20.0	<20.0	<20.0	<20.0	<20.0	<20.0	<20.0	<20.0	<20.0	<20.0	<20.0
		90	<20.0	<20.0	<20.0	<20.0	<20.0	<20.0	<20.0	<20.0	<20.0	<20.0	<20.0	<20.0	<20.0	<20.0	<20.0	<20.0	<20.0	<20.0
2	AA104aRH5	0	<20.0	<20.0	<20.0	<20.0	<20.0	<20.0	<20.0	<20.0	<20.0	<20.0	<20.0	<20.0	<20.0	<20.0	<20.0	<20.0	<20.0	<20.0
		90	<20.0	<20.0	<20.0	<20.0	<20.0	<20.0	<20.0	<20.0	<20.0	<20.0	<20.0	<20.0	<20.0	<20.0	<20.0	<20.0	<20.0	<20.0
2	AA107awg8	0	21	<20.0	<20.0	<20.0	<20.0	<20.0	<20.0	<20.0	<20.0	<20.0	<20.0	<20.0	<20.0	<20.0	<20.0	<20.0	<20.0	<20.0
		90	<20.0	<20.0	<20.0	<20.0	<20.0	<20.0	<20.0	<20.0	<20.0	<20.0	<20.0	<20.0	<20.0	<20.0	<20.0	<20.0	<20.0	<20.0
2	CNE8	90	<20.0	<20.0	<20.0	<20.0	<20.0	<20.0	<20.0	<20.0	<20.0	<20.0	<20.0	<20.0	<20.0	<20.0	<20.0	<20.0	<20.0	<20.0
2	CM244	90	<20.0	<20.0	<20.0	<20.0	<20.0	<20.0	<20.0	<20.0	<20.0	<20.0	<20.0	<20.0	<20.0	<20.0	<20.0	<20.0	<20.0	<20.0
1	Bal.26	23	27	41	38	29	50	<20.0	22	35	35	34	<20.0	21	<20.0	62	24	<20.0	32	23
		49	83	58	90	41	82	115	86	45	40	83	141	24	84	64	73	60	67	24
		90	61	<20.0	60	41	59	52	49	34	<20.0	66	204	36	127	43	50	62	35	34
1	6644.v2.c33	90	161	273	174	72	150	70	154	288	78	316	126	116	162	66	82	115	91	92
1	MN.3	23	430	805	1223	506	2071	1047	356	630	944	1168	172	159	216	898	469	280	534	367
		49	5582	3881	10462	2043	8671	9409	1677	2913	2465	7146	14489	1202	3162	2215	4386	12512	4471	1585
1	MW965.26	23	436	2737	1782	467	1176	617	1007	752	660	2054	96	183	376	451	508	409	955	349
		49	3796	21095	12579	1977	27131	4865	5421	6942	6789	18634	5048	1493	3059	2169	3176	12778	3071	1476
		90	6740	14642	13489	2364	7077	4173	12029	16107	2805	15825	11301	5734	10788	5093	3653	8430	3146	3677
1	NP03.13	23	70	140	91	<20.0	152	<20.0	34	108	28	117	24	69	343	39	92	36	132	112
		49	59	475	298	57	556	198	579	108	29	606	561	357	1363	160	264	258	488	257
		90	164	508	225	44	942	133	751	72	39	1727	2217	915	1605	427	832	898	1033	1273
1	SF162.LS	23	32	42	34	38	74	<20.0	29	61	32	41	<20.0	37	39	53	27	<20.0	42	48
		49	962	658	1417	588	1269	1025	568	796	281	2261	2070	81	777	499	807	931	378	329
		90	2352	458	1109	652	1304	1046	664	1127	215	3532	3513	311	1078	684	1363	1814	729	780
1	SS1196.1	90	64	32	46	31	57	62	61	46	<20.0	57	75	38	75	34	39	63	40	40
1	TH023.6	23	2665	2153	1963	922	2431	177	2609	3024	2105	8601	273	18492	3575	3223	5661	1217	2954	1425
		49	925	6575	4303	1044	4079	896	1199	2260	1280	3880	722	6924	3971	1579	2221	7328	2184	1044
		90	803	1294	1020	6818	5143	1303	10403	5740	5254	1727	2437	1702	18046	2807	2476	4900	2206	3161
Negative	SVA-MLV	49	<20.0	<20.0	<20.0	28	<20.0	<20.0	43	<20.0	<20.0	<20.0	<20.0	<20.0	<20.0	27	23	<20.0	22	<20.0
		90	<20.0	<20.0	<20.0	<20.0	<20.0	<20.0	<20.0	<20.0	<20.0	<20.0	<20.0	<20.0	<20.0	<20.0	<20.0	<20.0	<20.0	<20.0

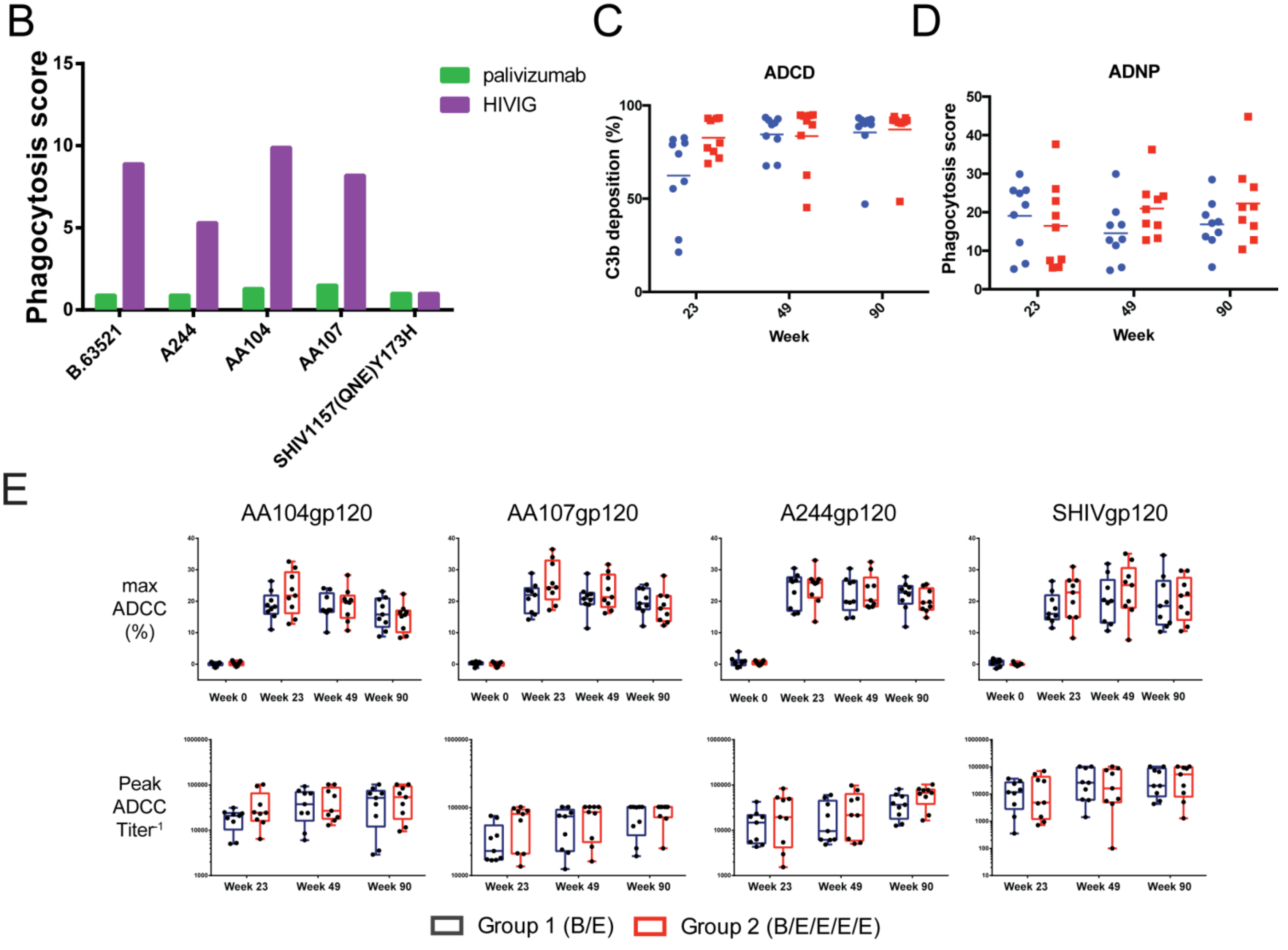

Supplementary Figure 4. Plasma antibody effector functions. (A) Plasma neutralization of Tier-1 viruses at weeks 23, 49 and prior to challenge at week 90 and the tier-2 viruses at week 90 measured in the TZM-bl neutralization assay (red > 500, orange > 50, yellow < 50). **(B)** Phagocytosis of vaccine and challenge gp120 coated targets by postivie (HIVIG) and negative (palivizumab) controls. Average of two replicate experiments. **(C-D)** High-througput Fc functional analysis. Purified plasma IgG from each animal from group 1 and 2 were tested for **(C)** Complement deposition (C3b) on the surface of A244 gp120 pulsed CD4+ target cells from HIV-negative donors. **(D)** Antibody-dependent Neutrophil-mediated phagocytosis against A244 gp120 coated beads by primary neutrophils. Horizontal line indicates group mean. **(E)** Max ADCC (top), peak ADCC titer (Endpoint dilution that scored above positive threshold; bottom) of gp120 coated target cells (CEM.NKR$_{CCR5}$) by plasma from both immunization groups measured using the ADCC-GTL assay.

A

#	Binding	Blocking	Neutralization	ADCP	ADCC
1	A244 gp120	A32 x A244 gp120	Bal.26	A244	Binding infected cells MFI (CM235 infected)
2	AA058 gp120	2g12 x JRFL	MW965.26	63521	Binding infected cells %cells (CM235 infected)
3	AA104 gp120	CH01 x A244 gp120	NP03.13	AA104	ADCC % killing (CM235 infected)
4	AA107 gp120	CH103 x 63521 gp120	SF162.LS	AA107	ADCC titer (CM235 infected)
5	63521 gp120	CH103 x CH505 TF gp120	SS1196.1	SHIV-1157(QNE)Y173H	ADCC titer (A244 gp120)
6	6644 gp120	CH58 x A244 gp120	TH023.6		ADCC titer (AA104gp120 coated)
7	63521 V2 peptide	CH59 x A244 gp120	GeoMean		ADCC titer (AA107gp120 coated)
8	A244 V2 peptide	CD4 x 63521 gp120			ADCC titer (SHIV1157(QNE)Y173Hgp120 coated)
9	AA058 V2 peptide	CD4 x A244 gp120			ADCC %GzB (CM235 infected)
10	AA104 V2 peptide	CD4 x CH505 T F gp120			ADCC %GzB (A244 gp120)
11	AA107 V2 peptide	CD4 x JRFL			ADCC %GzB (AA104gp120 coated)
12		PG9 x A244 gp120			ADCC %GzB (AA107gp120 coated)
13		PG9 x B 6240 gp120			ADCC %GzB (SHIV1157(QNE)Y173H gp120 coated)

B

HT-Functional Assays (A244 gp120)
ADCD
ADNP
ADCC
ADCP
MIP-1β
CD107a
IFN-γ

C

#	Binding	Detection Reagents
1	A244 gp120	Rh.IgA
2	AA058 gp120	Rh.IgG
3	AA104 gp120	Hu.C1q
4	AA107 gp120	Hu.FcgRIIAH
5	63521 gp120	Hu.FcgRIIAR
6	6644 gp120	Hu.FcgRIIB
7	63521 V2 peptide	Hu.FcgRIIIAF
8	A244 V2 peptide	Hu.FcgRIIIAV
9	AA058 V2 peptide	Hu.FcgRIIIB NA1
10	AA104 V2 peptide	Rh.FcgR2A-3
11	AA107 V2 peptide	Rh.FcgR2A-4
12	A244 V1V2 tags	Rh.FcgR3A-1
13	B.CaseA V1V2 tags	Rh.FcgR3A-3
14	SHIV-1157(QNE)Y173H gp120	
15	BAL gp120	
16	CM2235 gp120	
17	Du151 gp120	
18	Du156.12 gp120	
19	JRCSF gp120	
20	MN gp120	
21	PVO gp120	
22	YU2 gp120	
23	ZM109F gp120	
24	Du151 gp140	
25	HxBc2 gp41	
26	HxBc2 p24	
27	SHIV-SF162p3 V1V2	
28	A1.con_env03 gp140	
29	AE01.con_env03 gp140	
30	B.con_env03 gp140	
31	C.con_env03 gp140	
32	CON-S gp140	
33	ConB gp70 V1V2	
34	ConC gp70 V1V2	
35	SF162 gp120	
36	SF162.LS gp140	

D

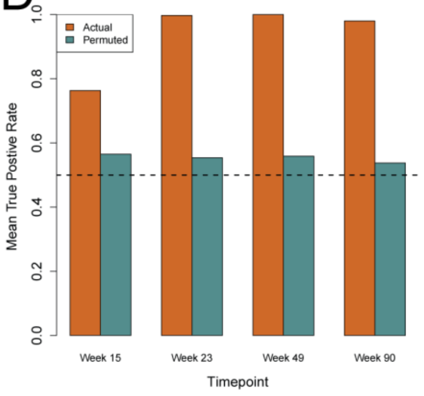

E

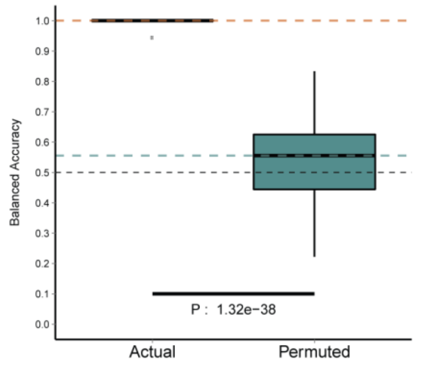

F

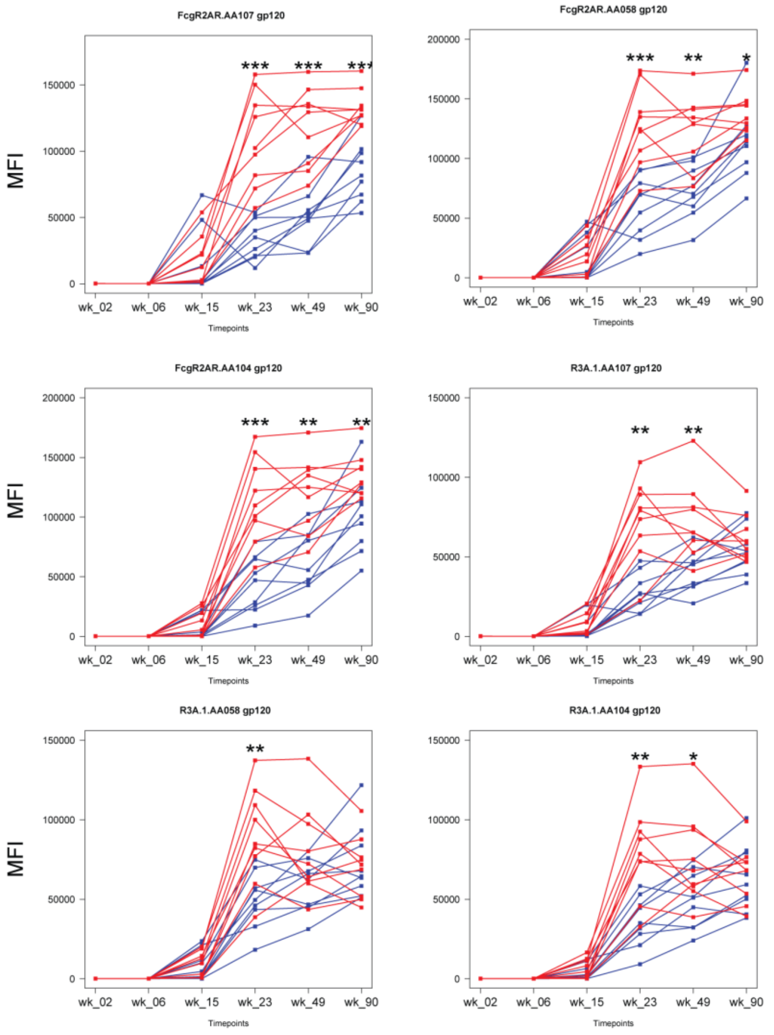

Supplementary Figure 5. Bivalent and pentavalent vaccine arms elicit different antibody responses. (A) Parameters that were measured pre-challenge (week 90) that were used for correlate analysis. **(B)** Parameters measured by high-throughput antibody profiling with A244 gp120 coated target cells. **(C)** Antigens and detection reagents used in the Fc array assay. **(D)** Mean True Positive Rate (balanced accuracy) of 100 runs of 9-fold cross-validation from weeks 15 through 90, comparing accuracy in predicting true group labels (cyan) vs. permuted labels (orange). The classifiers were trained on measurements taken only at the respective timepoints. **(E)** Test of robustness for models trained on measurements taken at the second protein boost (week 23), comparing performance of 100 repetitions of 9-fold cross-validation for actual (orange) vs. permuted (cyan) group labels. Significance was evaluated using the Wilcoxon-Mann-Whitney test. **(F)** Other antibody responses illustrating the evolving differences between groups; Wilcoxon-Mann-Whitney, * : P<0.05, ** : P<0.01, *** : P<0.001.

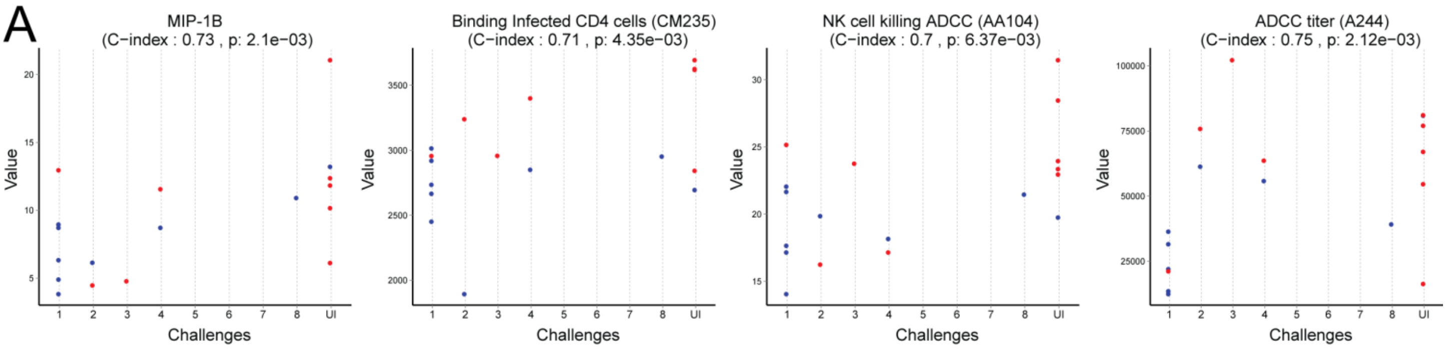

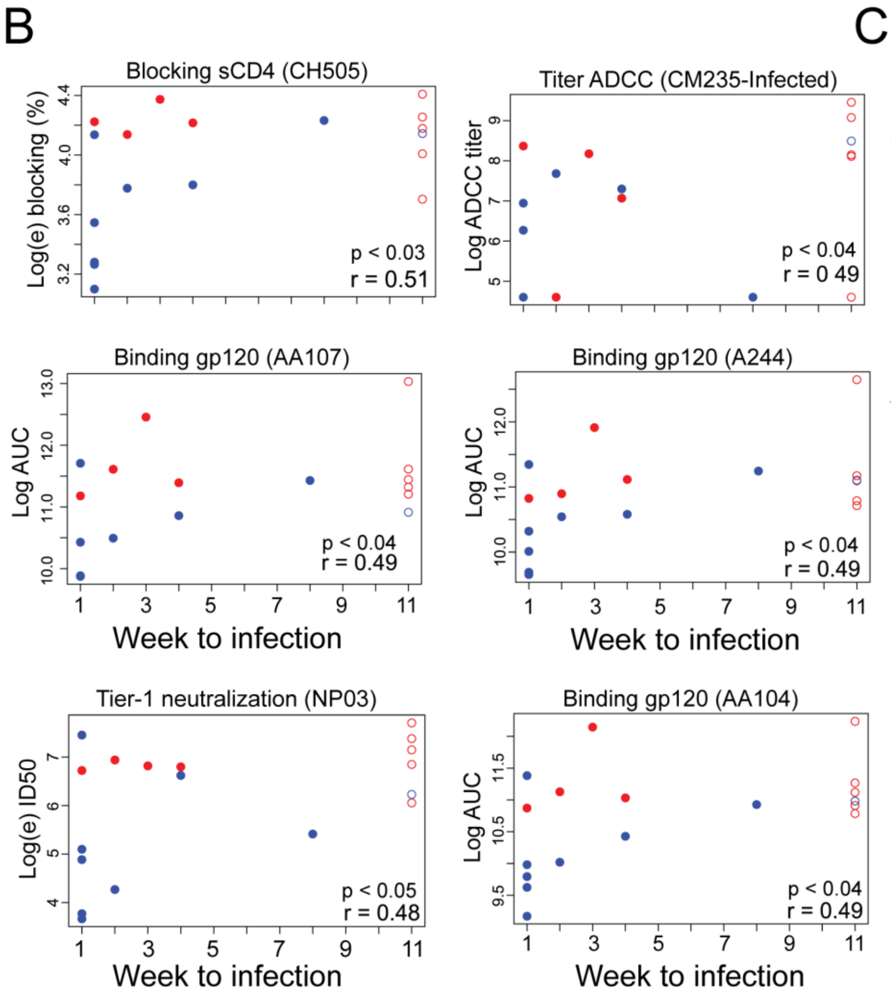

C

Parameter estimates from Cox Proportional Hazard model with time varying covariates.

Parameter	Hazard ratio	P-value
Binding B.63521 gp120	1.060	0.9051
Binding AA107 gp120	0.448	0.0769
Binding AA107 V2 peptide	0.961	0.8222
Blocking A32 on A244 gp120	0.094	0.3211
Blocking sCD4 on CH505 gp120	0.176	0.1568
Blocking sCD4 on JRFL gp120	0.882	0.5735

Supplementary Figure 6. Correlates of delayed infection. (A) C-indices of individual variables used in Fig. 5E. **(B)** Correlation of antibody ADCC, binding and blocking titers and tier-1 neutralization at week 90 with the number of weeks after challenge required to establish infection. The plotted data reflect only the vaccinated animals and not controls. Group 1 (B/E) in blue and group 2 (B/E/E/E/E) in red, open circles indicate animals uninfected after 8 challenges. *P* values reflect Spearman rank correlation tests. **(C)** Parameters that were measured pre-challenge (week 90) and weekly until infection. *P* values reflect analysis with a Cox Proportional Hazard model with time varying covariates.

A

Group	Animal	Week	Hook	# Antibodies
B/E/E/E/E	4312	23	B.63521 V1/V2	3
B/E/E/E/E	4312	23	A244 gp120 V. N156N160K	1
B/E/E/E/E	4812	23	A244 V1/V2	8
B/E/E/E/E	4812	23	A244 gp120	29
B/E/E/E/E	4812	49	A244 gp120	96
			**Total**	**137**

B

Antibody ID	VH gene	HCDR3 Length (AA)	VH Mutation (nt %)	VL gene	LHCDR3 Length (AA)	VL mutation (nt %)	Specificity
DH631	1-E	16	4.5	λ2-D	10	1.7	CD4bs
DH632	1-E	16	6.6	λ2-D	10	2	CD4bs
DH633	1-E	16	4.5	λ2-D	10	4	CD4bs
DH635	4-J	20	7.1	κ7-1	8	4.3	CD4bs
DH640	4-D	11	7.8	κ2-1	9	3.6	gp120
DH637	4-K	17	5.7	κ2-S4	9	3.5	V1/V2
DH638	4-F	16	8.9	λ5-B	9	1.9	V1/V2
DH641	3-AA	17	4.4	λ2-F	10	6	V1/V2

C

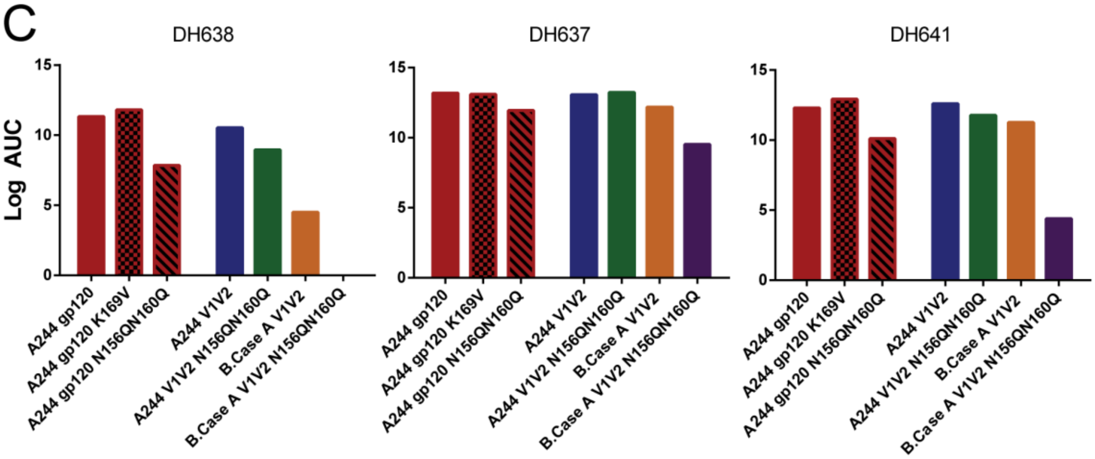

D

Peptide	Clade/Strain	Sequence	Signal Intensity
56	A244&TH023	VHALFYKLDIVPIED	6,273
56	MN	EYALLYKLDIEPIDN	3,327
56	1086	VHALFYKLDVVPLNG	4,529
56	TV1	EYALFYRLDIVSLNN	6
56	ZM651	VYALFYKLDIVSLNE	5
56	M	VYALFYRLDVVPINN	9
56	A	VYSLFYRLDVVQINE	160
56	B	EYALFYKLDVVPIDN	2,575
56	C	VYALFYRLDIVPLNE	6
56	D	VYALFYKLDVVPIDN	23
56	CRF1	VYALFYKLDIVQIEN	398
56	CRF2	VYALFYRLDVVQINE	1
57	A244	LFYKLDIVPIEDNND	7,979
57	A244	YKLDIVPIEDNNDSS	24,232
57	TH023	LFYKLDIVPIEDNTS	3,169
57	TH023	YKLDIVPIEDNTSSS	23,156
57	MN	LLYKLDIEPIDNDST	2,300
57	1086	LFYKLDVVPLNGNSS	12,100
57	1086	KLDVVPLNGNSSSSG	1,284
57	1086	DVVPLNGNSSSSGEY	1,321
57	TV1	LFYRLDIVSLNNKPN	3,511
57	ZM651	LFYKLDIVSLNETDD	49
57	M	LFYRLDVVPINNNSS	665
57	A	LFYRLDVVQINENSN	11,677
57	B	LFYKLDVVPIDNDNT	8,932
57	C	LFYRLDIVPLNENNS	5,076
57	D	LFYKLDVVPIDNNNN	5,483
57	CRF1	LFYKLDIVQIENNNS	11,604
57	CRF2	LFYRLDVVQINENNS	1,086

E

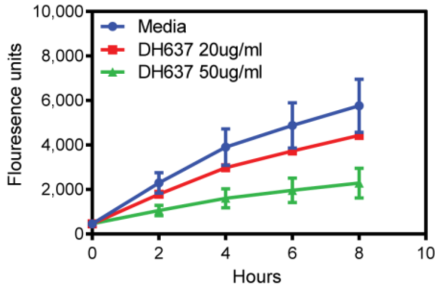

α4β7 inhibition (%)	Experiment 1		Experiment 2	
Antibody	20μg/ml	50μg/ml	20μg/ml	50μg/ml
DH637	42.2	65.2	23.1	60.3
Ab82	7.7	25.3	0	3.4
7B2	0	7.7	6.7	19.1

F

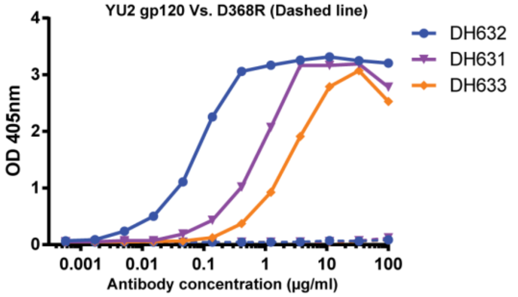

Supplementary Figure 7. Isolation of antibodies from macaques immunized with the pentavalent vaccine. **(A)** Number of antibodies isolated from each animal, timepoint and antigen used for sorting. **(B)** Immunogenetic characteristics of isolated antibodies. **(C)** ELISA binding of DH638, DH637 and DH641 to wild-type and mutated gp120 and V1/V2 HIV antigens. **(D)** DH637 binding (signal intensity) to peptides on a linear microarray. Sequences of peptides 56 and 57 that are in the V2 and contain the $\alpha_4\beta_7$ integrin binding site for each clade/strain are shown. **(E)** Inhibition of α4β7 on RPMI8866 cells to cyclic 92TH023 (Clade E Env) V2 epitope peptide by DH637. Representative graph of blocking over time of α4β7 by DH637 at 20μg/ml and 50μg/ml antibody concentrations or media (blue) over time. Chart below shows the percent inhibition of both experiments by DH637 and two negative control antibodies (Ab82, influenza; 7B2, HIV gp41). Both Experiments performed in triplicate. Error bars indicate standard deviation. **(F)** ELISA binding of DH632, DH631 and DH633 to YU2 gp120 (Solid line) and YU2 gp120 D368R mutant (Dashed line).
